# Aromatic Amino Acids: Exploring Microalgae as a Potential Biofactory

**DOI:** 10.3390/biotech14010006

**Published:** 2025-01-29

**Authors:** Archana Niraula, Amir Danesh, Natacha Merindol, Fatma Meddeb-Mouelhi, Isabel Desgagné-Penix

**Affiliations:** Department of Chemistry, Biochemistry and Physics, Université du Québec à Trois-Rivières, Trois-Rivières, QC G8Z 4M3, Canada; archana.niraula@uqtr.ca (A.N.); amir.danesh@uqtr.ca (A.D.); natacha.merindol@uqtr.ca (N.M.); fatma.meddeb@uqtr.ca (F.M.-M.)

**Keywords:** shikimate pathway, *Chlamydomonas reinhardtii*, chorismate mutase, *Phaeodactylum tricornutum*, metabolic engineering

## Abstract

In recent times, microalgae have emerged as powerful hosts for biotechnological applications, ranging from the production of lipids and specialized metabolites (SMs) of pharmaceutical interest to biofuels, nutraceutical supplements, and more. SM synthesis through bioengineered pathways relies on the availability of aromatic amino acids (AAAs) as an essential precursor. AAAs, phenylalanine, tyrosine, and tryptophan are also the building blocks of proteins, maintaining the structural and functional integrity of cells. Hence, they are crucial intermediates linking the primary and specialized metabolism. The biosynthesis pathway of AAAs in microbes and plants has been studied for decades, but not much is known about microalgae. The allosteric control present in this pathway has been targeted for metabolic engineering in microbes. This review focuses on the biosynthesis of AAAs in eukaryotic microalgae and engineering techniques for enhanced production. All the putative genes involved in AAA pathways in the model microalgae *Chlamydomonas reinhardtii* and *Phaeodactylum tricornutum* are listed in this review.

## 1. Introduction

Microalgae are microscopic photosynthetic primary producers in marine ecosystems that are responsible for a significant portion of global CO_2_ sequestration. They are adaptable to varying conditions, displaying a wide array of genetic diversity and metabolic capabilities that can be harnessed [1,2]. In addition to their ecological importance, microalgae are also rich in valuable components, such as essential unsaturated fatty acids, pigments, amino acids, and antioxidants, making them attractive resources for various industries, including pharmaceuticals, cosmetics, food, and biofuel production [3]. Furthermore, their use for cost-effective, sustainable production systems and their potential as platforms for heterologous protein expression make them a promising resource for biotechnological innovation [4,5,6]. Among various microalgae, this review focuses on the green alga *Chlamydomonas reinhardtii* and the marine diatom *Phaeodactylum tricornutum* due to their promising features as a model organism for both fundamental studies and as a biotechnological platform. Their ability to produce therapeutic proteins, antibiotics, antigens, carotenoids, polyunsaturated fatty acids, and cytochrome P450s has been widely explored [7,8,9,10,11,12]. Despite the many molecular tools and resources that have been developed, there are still challenges to their successful engineering, particularly in obtaining reproducible and high levels of stable transgene expression. Some examples of challenges include low transgene expression levels, transgene instability, low productivity in large-scale cultivation, metabolic flux management, post-translational modifications (PTMs), limited genetic tools and resources, transformation efficiency, gene silencing, etc. [13]. In this section, the focus will be on the intriguing features and industrial potential of *C. reinhardtii* and *P. tricornutum*.

### 1.1. Chlamydomonas Reinhardtii

*Chlamydomonas reinhardtii* (in Greek *chlamys*, a cloak; *monas*, solitary), is a unicellular green algae species used as a model organism and, in recent times, as a host for biotechnological interventions [14]. It is a photosynthetic biflagellate microalga of 5–10 µm in diameter that belongs to the *Chlorophyceae* class. Each cell wall is composed of hydroxyproline-rich glycoprotein, and the cell contains a single large cup-shaped chloroplast (occupying two-thirds of cell volume), a centrally located nucleus with a distinct nucleolus, two anterior flagella, and several mitochondria. [15]. *C. reinhardtii* can grow in media containing complex carbon sources, such as acetate under photoautotrophic, mixotrophic, or heterotrophic conditions [16]. Compared to *Spirulina* and *Chlorella* for nutritional supplement purposes, *C. reinhardtii* has a higher potential to become a popular superfood due to its nutritional content, including 46.9% protein, 24.7% lipid content, 23.6% carbohydrates, and 4.8% ash content [17]. *C. reinhardtii* can be modified for the heterologous production of recombinant protein using its three sets of genomes (nuclear, mitochondrial, and plastid) [18]. It can process complex eukaryotic proteins through post-translational modifications, giving it an advantage over microbial host systems [19]. In addition, this microalga is generally recognized as safe by the FDA, which is an asset for recombinant protein production [20]. Research work using *C. reinhardtii* as a laboratory organism began more than 60 years ago [21]. Over time, *C. reinhardtii* has emerged as a prominent model in various realms of cellular and molecular biology. Its nuclear genome is GC-rich, is estimated to contain 17,741 genes, and is about 101.1 Mb in size [19]. The chloroplast genome is roughly 203 kb, containing 99 genes [16], while the mitochondrial genome is linear and 15.8 kb in size, containing eight genes [22]. Expression from the chloroplast genome offers several advantages due to its size and ease of genetic manipulation, allowing for precise targeting of transgenes to any preferred locus for regulated and stable expression [23]. Research has led to the development of effective endogenous and synthetic promoters, improved transformation methods, codon optimization/intron insertion tools, and a variety of selectable markers, which have significantly increased the efficiency of exogenous DNA transfer into the nucleus of *C. reinhardtii* [14,20,22,24,25,26,27,28]. These advanced tools, developed over the past few decades, have facilitated many metabolic-engineering strategies to produce desired compounds, as listed in Table 1.

### 1.2. Phaeodactylum Tricornutum

Another emerging model of microalga is the pennate marine diatom *Phaeodactylum tricornutum*, one of the most studied and commercially suitable species for large-scale cultivation, which has been described as a ‘‘diatom cell factory’’ [36]. *P. tricornutum* can be found growing in brackish-to-saline water in various locations around the world and exhibits a unique polymorphic nature, displaying three principal morphotypes: oval, triradiate, and fusiform [37]. Due to changes in growth conditions, such as salinity, pH, and temperature, cells can undergo reversible morphological transitions from one form to another.

Under typical cultivation conditions, on average, the biomass of this species contains 36.4% crude protein, 26.1% carbohydrates, 18.0% lipids, and 15.9% ash on a dry weight basis [38]. *P. tricornutum* is particularly appealing due to its small genome size, fast growth (doubling times typically in hours and biomass productivity in days), and ease of genetic manipulation [36]. This diatom has emerged as a prominent model for investigating photosynthesis and lipid metabolism, with numerous studies over the past decade aimed at enhancing the biosynthesis of fatty acids, triacylglycerols (TAG), and polyunsaturated fatty acids (PUFA) [39,40,41,42,43].

*P. tricornutum* is considered the first diatom species to be successfully genetically engineered. Bowler et al. [44] sequenced and annotated the genome of *P. tricornutum* CCAP 1055/1 in 2008, which was estimated to contain 10,402 protein-encoding genes with a size of approximately 27.4 Mbp. Its genome was revised in 2018 [45], 2021 [46], and 2022 [47]. Additionally, approximately 130,000 expressed sequenced tags have been reported for *P. tricornutum*. The development of an extensive genetic toolkit, such as the identification of endogenous promoters, well-mapped plasmids, antibiotic-free selection markers, and successful DNA delivery methods, has facilitated genetic manipulations, such as gene overexpression, silencing, and editing, highlighting the versatility of *P. tricornutum* in biotechnological applications [39,40,41,42,43]. *P. tricornutum* is a promising candidate for metabolic engineering aimed at developing a platform for the production of pharmaceutically relevant metabolites [41,48]. Moreover, advances in genetic knowledge of *P. tricornutum* have contributed to its development for the production of non-native components, including polyhydroxybutyrate (PHB) for bioplastics [49], monoclonal antibodies [50], plant triterpenoids [12], and cannabinoids [40]. Several experimental approaches for natural product accumulation and recombinant protein production have been applied to *P. tricornutum,* with a few examples listed in Table 2.

### 1.3. Aromatic Amino Acids

Aromatic amino acids (AAA), phenylalanine (L-Phe), tyrosine (L-Tyr), and tryptophan (L-Trp), are essential building blocks for proteins and are involved in various physiological processes of living organisms. These amino acids are essential for maintaining the structural and functional integrity of proteins in cells [62]. They are involved in vital biological processes, such as signal transduction, cell growth and cell division, DNA replication, and defense mechanisms [63]. AAAs have been found to be involved in the stress response in *C. reinhardtii*, such as stress caused by methanol supplementation [64], hyperosmotic stress [65], and the application of sublethal concentrations of mercury (Hg) [66], which enhances L-Tyr and L-Phe. In *Chlorophyta* spp., the introduction of the quorum-sensing molecule N-acylhomoserine lactone, extracted from activated sludge bacteria increased the production of AAAs and the formation of cell aggregates [67].

L-Phe is a precursor of more than 8000 plant phenolic compounds, including the phytohormone salicylic acid, quinones (ubiquinones), attracting compounds (anthocyanins and phenylpropanoid/benzenoid volatiles), phytoalexins, feeding deterrents (tannins), and defense and UV protecting (flavonoids and various phenolics), signaling (isoflavonoids), and structural components (lignin, suberin, and cell wall-associated phenolics). The broad physiological functions of these compounds explain the high carbon flux through the AAA metabolic network. In vascular plants, up to 30% of photosynthetically fixed carbon is directed toward L-Phe biosynthesis for the production of abundant phenylpropanoid compounds [68]. Likewise, L-Phe and the L-Tyr pathway intermediate 4-hydroxyphenylpyruvate serve as precursors for numerous specialized metabolites that are crucial for both plant and human health, such as the antioxidant vitamin E, the photosynthetic electron carrier plastoquinone, the betalain pigments, and defense compounds, including dhurrin, rosmarinic acid, and alkaloids (e.g., morphine) [69]. In addition, compounds such as the monoamine neurotransmitters serotonin, dopamine, epinephrine, and norepinephrine, which are found in the central and peripheral neural systems of most mammalian species, are biosynthesized from L-Tyr [70]. L-Trp also serves as a starting material to produce pharmaceuticals, such as antidepressants and antitumor drugs. In plants, L-Trp acts as a precursor for indole alkaloids, indole glucosinolates, and phytoalexins, all playing a vital role in defense mechanisms, growth, regulation, pollination, etc. [71].

Thus, the biosynthesis of AAAs in plants serves a multifaceted purpose, including a critical role in the production of specialized metabolites. In this context, enhancing the levels of AAA precursors is a key strategy to increase the production of specialized metabolites [43,72]. Plants, bacteria, fungi, and certain protists can synthesize the essential AAAs necessary for their biological processes, whereas animals, including humans, lack this capability. Consequently, animals must obtain these essential amino acids from their diet to maintain proper biological function [73].

Our understanding of AAA biosynthesis in microalgae is limited, but most of their metabolic pathways can be inferred from the presence of transcripts orthologous to microbial and higher plant enzymes. The Section 2 outlines our current understanding of AAA biosynthesis pathways, with insights inferred from the presence of the corresponding transcripts in microalgae, suggesting potential transposition of knowledge. Additionally, the localization of these enzymes was predicted using the software tools ‘WoLF PSORT II protein localization prediction tool’ (https://wolfpsort.hgc.jp, accessed on 28 January 2025) and ‘ProtComp 9.0 protein localization prediction tool’ (Table A1 and Table A2). However, the prediction results are conflicting for most of the enzymes, indicating the importance of further experimental validation.

## 2. Biosynthesis of AAA

### 2.1. Shikimate/Chorismate Pathway

**Figure 1:**
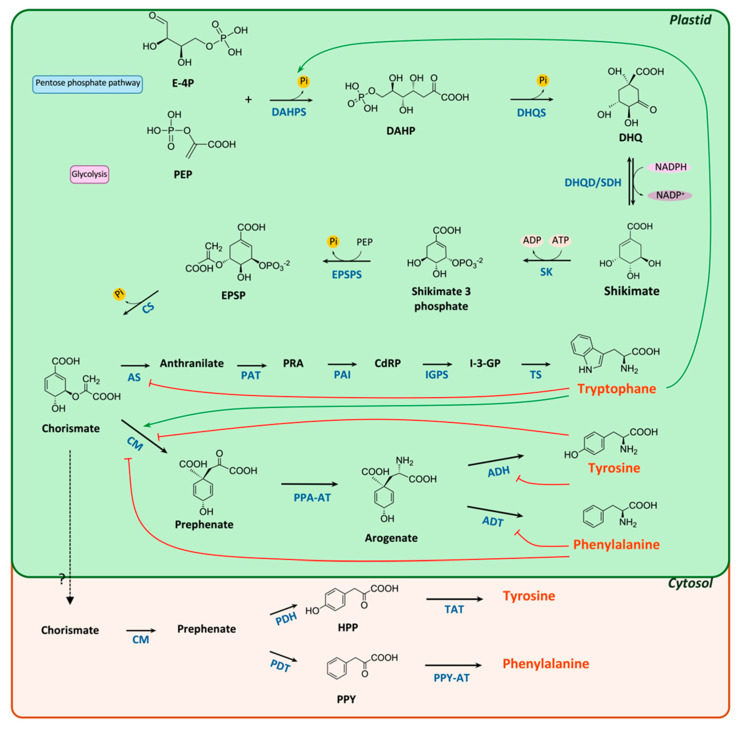
Proposed aromatic amino acid (AAA) biosynthesis in *P. tricornutum* and *C. reinhardtii*. Black arrows represent enzymatic steps supported by direct experimental evidence. Solid red blocks represent known feedback inhibition; green arrows represent known feedback activation. Not all shown enzymes may be present in all plant species. Abbreviations: PEP, phosphoenolpyruvate; E-4P, erythrose 4-phosphate; DAHPS, 3-deoxy-d-arabinoheptulosonate 7-phosphate (DAHP) synthase; DHQS, 3-dehydroquinate (DHQ) synthase; DHQD, 3-dehydroquinate dehydratase; SDH, shikimate 5-dehydrogenase; SK, shikimate kinase; EPSPS, 5-enolpyruvylshikimate 3-phosphate (EPSP) synthase; CS, chorismate synthase; AS, anthranilate synthase; PAT, phosphoribosylanthranilate transferase; PRA, 5-phosphoribosylanthranilate; PAI, phosphoribosylanthranilate isomerase; CdRP, 1-(o-carboxyphenylamino)-1-deoxy-ribulose 5-phosphate; IGPS, indole-3-glycerol phosphate synthase; I-3-GP, indole-3-glycerol phosphate; TS, tryptophan synthase; CM, chorismate mutase; PPA-AT, prephenate aminotransferase; ADH, arogenate dehydrogenase; ADT, arogenate dehydratase; PDH, prephenate dehydrogenase; PDT, prephenate dehydratase; PPY-AT, phenylpyruvate aminotransferase; TAT, Tyr aminotransferase; Pi, inorganic phosphate.

The shikimate pathway plays a crucial role in linking central carbon metabolism with the AAA network. It leads to the biosynthesis of AAAs from basic primary metabolites through a series of seven enzymatic reactions, converting phosphoenolpyruvate (PEP) and _D_-erythrose 4-phosphate (E4P), metabolic intermediates derived from glycolysis and the pentose phosphate pathway (PPP), respectively, into chorismate, the main precursor [74]. An aldol condensation of PEP and E4P catalyzed by 3-deoxy-_D_-arabino-heptulosonate-7-phosphate synthase (DAHPS/DHS; EC 2.5.1.54) initiates the first step to obtaining DAHP and inorganic phosphate, as shown in Figure 1. DAHPS requires a divalent metal ion cofactor, such as Zn^2+^, Cu^2+^, or Fe^2+^ [75,76]. A comparison of DAHPS’s kinetic parameters within microbial and plant systems reveals a higher *K_m_* value for E4P than PEP, suggesting less affinity between E4P and DAHPS (Table 3). Control of the carbon flow towards AAA biosynthesis in microbes is known to be primarily controlled by feedback inhibition of DAHPS at this step [77], whereas in plants, the regulation is quite complex and depends also on cofactor/substrate availability, redox regulation within plastids, and transcriptional regulation [78]. DAHPS is classified as type I or type II, and its representatives have less than 10% amino acid sequence similarity [68]. DAHPS type I enzyme is found mostly in microbes, such as *E. coli*, *Saccharomyces cerevisiae*, and *Bacillus subtilis*, whereas the type II enzyme occurs mostly in plant species, as well as in certain bacteria such as *Mycobacterium tuberculosis*, and *Neurospora crassa* [79]. The type I enzyme is further subdivided into Iα and Iβ based on the regulatory regions and catalytic core [76]. Each of these subtypes, Iα and Iβ, can be further divided based on allosteric regulation by either L-Phe, L-Tyr, L-Trp, chorismate, or prephenate. In *E. coli,* three isozymes of type Iα DAHPS exist, and each is regulated by either tyrosine (AroF), phenylalanine (AroG), or tryptophan (AroH) (Table 3) [80,81].

Type Iβ contains an N/C terminal extension that determines whether it is regulated by chorismate/prephenate or tyrosine/phenylalanine [87]. The enzyme localization was previously known to be in the plastid. However, recent research in *A. thaliana* revealed cytosolic retention of DHS2 as well [88]. In the *C. reinhardtii* database, there are three candidates, Cre17.g726750_4532.1, Cre17.g726750_4532.2, and Cre17.g726750_4532.3, obtained from the basic local alignment search tool (BLAST) with characterized sequences (Table A1). The resulting sequences are predicted to be localized to the chloroplast, as per the software ‘WoLF PSORT II protein localization prediction tool’ and ‘ProtComp 9.0 protein localization prediction tool’, and belong to the type II DAHPS classification. Additionally, in *P. tricornutum*, one candidate for DAHPS, XP_002177054.1, was predicted to localize in the chloroplast (Table A2). Research on the carbon flow through DAHPS towards AAA biosynthesis in microalgae has yet to be performed. The second step of the shikimate pathway consists of the cyclization of DAHP to 3-dehydroquinate (3-DHQ) catalyzed by DHQ synthase (DHQS; EC 4.2.3.4) [89] (Figure 1). DHQS requires two cofactors: a divalent cation for precise and effective substrate binding and NAD^+^, which is required for substrate oxidation at the first step of the reaction [74]. The enzyme functions as a monomer, except for fungi, where this enzyme is part of the pentafunctional complex (AroM) [79,90]. In the microalgae *Euglena gracilis*, the AroM complex is present, similar to fungi, and carries out intermediate steps to form 5-enolpyruvyl-shikimate 3-phosphate (EPSP). In *C. reinhardtii*, BLAST with a characterized sequence revealed two candidates, Cre08.g368950_4532.1 with predicted chloroplast localization and Cre08.g368950_4532.2 with predicted cytosol localization (Table A1). Additionally, in *P. tricornutum*, one candidate for DHQS, XP_002180805.1 (Phatr3_J20809), was predicted to localize in the chloroplast (Table A2).

The third and fourth steps consist of the dehydration of 3-DHQ to yield 3-dehydroshikimate (3-DHS), catalyzed by DHQ-dehydratase (DHQD, EC 4.2.1.10), and the reduction of 3-DHS into shikimate in presence of shikimate dehydrogenase (SDH; EC 1.1.1.25) (Figure 1). DHQD can be classified as type 1 and type II, both having distinct reaction mechanisms. Type I catalyzes a cis-dehydration reaction via covalent imine intermediates, whereas type II has weak covalent intermediates and catalyzes a trans-dehydration reaction through enolate intermediates [74,91]. Type I DHQD is found in plants, fungi, and many bacterial species for the biosynthesis of chorismate, while type II is particular to the quinate and shikimate pathway in fungi and some bacteria, respectively [92]. DHQD/SDH-like genes are involved in cold stress adaptation in the freshwater alga *Spirogyra varians* [93] and in response to mechanical wounding in plants [94]. In microalgae and plants, the enzymes DHQD and SDH are fused to form a DHQD/SDH bifunctional complex, whereas, in *E. coli*, they act as monofunctional enzymes (AroD for DHQ and AroE for SDH, respectively) [95,96]. In fungi, these enzymes are a part of the pentafunctional AroM complex [90]. In *C. reinhardtii*, BLAST with a characterized sequence revealed one candidate, Cre08.g380201_4532.1, with the predicted chloroplast localization (Table A1). In *P. tricornutum*, one candidate for DHQ/SDH, XP_002179655.1, was predicted to localize in the chloroplast (Table A2).

In the fifth step, shikimate is converted to shikimate-3-phosphate in the presence of shikimate kinase (SK; EC 2.7.1.71) using ATP as a co-substrate [97]. As SK is the only known enzyme for the phosphorylation of the C3 hydroxyl group of shikimate to give 3-phosphate shikimate (S3P), it is essential for the further biosynthesis of 5-enolpyruvyl-shikimate 3-phosphate (EPSP) (Figure 1) [98]. Two SK genes are present in *E. coli,* namely *AroK* and *AroL*. The latter is known to play a vital role in chorismate biosynthesis [74]. In plants, numerous isoforms of SK are present, for instance, two in *A. thaliana* and three in *Oryza sativa*, and they all localize in the chloroplast [68]. In algae, lycophytes, and bryophytes, one SK gene can be found [99]. In *C. reinhardtii*, BLAST with a characterized sequence revealed two candidates, Cre10.g436350_4532.1, with predicted chloroplast localization, and another Cre10.g436350_4532.2 candidate with extracellular localization (Table A1). Meanwhile, in *P. tricornutum*, one candidate for SK, XP_002184173.1 (Phatr3_J6807), was predicted to localize in the chloroplast (Table A2).

The penultimate step of the shikimate pathway consists of the condensation of a second PEP with S3P to yield EPSP and inorganic phosphate (Figure 1). This reaction is catalyzed by the EPSP synthase (also known as 3-phosphoshikimate 1-carboxyvinyltranferase; EC 2.5.1.19), which is the well-known target for the herbicide glyphosate [100]. The isoforms of this enzyme are often classified according to their sensitivity to glyphosate. Class I, which includes all plants and most bacteria, is the most sensitive, compared to class II from some bacterial species (e.g., *Agrobacterium* sp. strain CP4), which is relatively resistant [101]. Interestingly, green algae, lycophytes, and bryophytes display one isoform, whereas plants encompass different numbers of EPSP synthase isoforms [99]. In *C. reinhardtii*, BLAST with a characterized sequence revealed one candidate, Cre03.g181300_4532.1, with the predicted chloroplast localization (Table A1), and similarly, for *P. tricornutum*, one candidate for EPSP synthase, XP_002178032.1, was predicted to localize in the chloroplast (Table A2).

The final step in the shikimate pathway consists of the introduction of the second double bond during the formation of chorismate by the *trans*-1,4 elimination of phosphate from EPSP, catalyzed by the chorismate synthase (CS; EC 4.2.3.5) (Figure 1) [102]. This reaction requires flavin mononucleotide (FMN) in its reduced form as a cofactor. Two functional types of CS have been identified using the ability to reduce the oxidized FMN as a criterion: monofunctional CS, which is present in the majority of bacteria and plants, and bifunctional CS, which is primarily found in fungi [74]. While fungal CS enzymes are linked to an NADPH-dependent flavin reductase, plants, bacteria, and other organisms depend on an external source of reduced FMN [103]. EPSP receives an electron from the FMN, which facilitates the cleavage of phosphate [98]. In the Antarctic green alga *Chlamydomonas* sp. UWO 241, experimental evidence shows the involvement of DAHPS along with CS in the adaptation of alga to high salt concentrations [104]. In *C. reinhardtii*, BLAST with a characterized sequence revealed two candidates, Cre03.g145747_4532.1 and Cre03.g145747_4532.2, with the predicted chloroplast localization (Table A1). Additionally, in *P. tricornutum*, one candidate for CS, XP_002177933.1 (Phatr3_J43429), was predicted to localize in the chloroplast (Table A2). For decades, the shikimate pathway has been known to function in the chloroplasts of plants. However, molecular evidence for the genes involved in this pathway, namely DAHPS, DHQ/SDH, and SK, with localization in the cytosol, has led to the theory of a dual shikimate pathway, as summarized in a recent review [71]. According to protein localization software, the candidates obtained for most of the enzymes in the *C. reinhardtii* and *P. tricornutum* database indicate chloroplast localization, suggesting that the shikimate pathway exists exclusively in the chloroplast.

### 2.2. Post-Chorismate Pathway

In the first step of the post-chorismate pathway, chorismate is converted to prephenate by chorismate mutase (CM), whose subsequent conversion to L-Phe or L-Tyr may occur via two alternative pathways (Figure 1). In one route (the plastidial or arogenate pathway), prephenate is first transaminated to L-arogenate, followed by dehydration/decarboxylation to L-Phe or dehydrogenation/decarboxylation to L-Tyr catalyzed by arogenate dehydratase (ADT) or arogenate dehydrogenase (ADH), respectively. In the alternative pathway (the cytosolic or phenylpyruvate pathway), the reactions occur in reverse order. Prephenate is first subjected to dehydration/decarboxylation by prephenate dehydratase (PDT) or to dehydrogenation/decarboxylation by prephenate dehydrogenase (PDH), followed by transamination of the corresponding products, phenylpyruvate and 4-hydroxyphenylpyruvate, to L-Phe or L-Tyr, respectively (Figure 1) [105].

### 2.3. Phenylalanine and Tyrosine Biosynthesis

#### 2.3.1. Chorismate Mutase (CM)

Chorismate, derived from the shikimate pathway, serves as a key precursor for the biosynthesis of diverse aromatic compounds, including AAAs. Chorismate mutase (CM; EC 5.4.99.5) catalyzes the initial step in the biosynthesis of tyrosine (L-Tyr) and phenylalanine (L-Phe) through a pericyclic Claisen rearrangement of the enolpyruvate side chain of chorismate to form prephenate [106,107]. In prokaryotes, two kinds of CMs have been identified based on protein folding: one with an α-helical structure (AroQ) and the other with an α/β-barrel structure (AroH) [68]. Representative of the AroH class of CMs, as found in *Bacillus subtilis*, are monofunctional non-allosteric enzymes, which function as a homotrimer. AroQ-type CMs are dimers, in which each monomer consists of approximately 250 amino acids, while AroH-type CMs are smaller dimeric structures with monomers composed of ~110 amino acids [108]. Additionally, there is an effector site within the regulatory region of AroQ-type CMs that regulates enzymatic activity [109]. This enzyme is active either as a monofunctional protein (with or without allosteric regulation) or as a bifunctional complex, such as CM-prephenate dehydratase (CM-PDT), CM-prephenate dehydrogenase (CM-PDH) in *E. coli*, and CM-DAHP synthase in *B. subtilis* [106,110].

Eukaryotic CMs from plants and fungi form a separate subclass of AroQ-type enzymes. These typically include two isozymes, CM1 and CM2, which are characterized by differences in subcellular localization and regulatory properties. CM1 is plastid-localized and exhibits allosteric regulation, being inhibited by L-Phe and L-Tyr and activated by L-Trp. In contrast, CM2 lacks a plastid transit peptide, is primarily localized in the cytosol, and is not subject to such regulation [111]. Allosteric CMs undergo different conformational states that are essential for their function. Initially, two allosteric states were described for the yeast CM1, namely R (active) and T (inactive) [112]. Recent NMR studies suggest that an additional super-active state (super-R) exists, playing a significant role in allosteric regulation [109,113,114].

In contrast to prokaryotes, recent genetic evidence indicates that the plastidial pathway is the predominant route for L-Phe and L-Tyr biosynthesis in plants [115]. The discovery of cytosolic phenylpyruvate aminotransferase (PPY-AT) and the presence of cytosolic CM in various plant species suggest that a complete pathway for L-Phe and L-Tyr biosynthesis exists in the cytosol [116,117], in addition to the established plastidial pathway [118,119]. Understanding of the cytosolic biosynthetic pathway remains limited, with only a few genes identified. The origin of cytosolic chorismate remains unclear; it may be transported out of the plastids via an unknown transporter or synthesized through a hypothetical, yet-to-be-discovered cytosolic shikimate pathway [71]. These uncertainties highlight the potential for new metabolic-engineering strategies to enhance the production of AAAs or related compounds in plants. Further research is required to elucidate the regulation and the full scope of the cytosolic AAA biosynthetic pathway [120].

CMs from various organisms, including plants, mosses, and yeasts (*Arabidopsis thaliana*, *Petunia hybrida*, *Physcomitrella patens*, *Hansenula polymorpha*, and *Aspergillus nidulans*), have been identified and biochemically characterized (Table 4). For instance, *A. thaliana* has three isoforms: AtCM1 (plastid localized and feedback inhibited by L-Phe and L-Tyr, and up-regulated by L-Trp), AtCM2 (cytosolic and non-regulated), and AtCM3 (plastid localized and activated by L-Trp, histidine, and cysteine) [111]. Similar trends are observed in *P. hybrida*, where PhCM1 is plastid-localized and regulated by L-Trp, while PhCM2 is cytosolic and non-allosteric [121]. Comparative sequence analyses reveal a conserved domain with minimal variation in effector-binding sites across plant taxa [108]. Additionally, basal plant lineages, such as bryophytes, lycophytes, and basal angiosperms, form a distinct phylogenetic clade compared to the isoform diversity observed in flowering plants [111]. In *C. reinhardtii*, a BLAST analysis identified three CM candidates, Cre03.g155200_4532.1 and Cre03.g155200_4532.2 with the predicted chloroplast localization, whereas Cre03.g155200_4532.3 had the predicted cytosol localization (Table A1). In *P. tricornutum*, two CM candidates, Phatr3_draftJ417 and Phatr3_J43277, were predicted to localize to both the chloroplast and cytosol (Table A2).

#### 2.3.2. Prephenate Aminotransferase

The prephenate aminotransferase (PPA-AT; EC 2.6.1.1) plays a crucial role in the plastidial pathway of L-Phe/L-Tyr biosynthesis, facilitating a reversible transamination between prephenate and arogenate using pyridoxal 5′-phosphate (PLP) as a cofactor (Figure 1). Plants contain more than one isoform of PPA-AT, whereas green algae contain a single gene [99]. PPA-AT has been identified in various plant species and some bacteria and exhibits a higher affinity for prephenate compared to phenylpyruvate or 4-hydroxyphenylpyruvate, two keto acid intermediates involved in L-Phe/L-Tyr biosynthesis. These enzymes utilize L-glutamate or L-aspartate as amino donors. Kinetic analysis of plant PPA-AT enzymes has revealed that their affinity for prephenate is approximately 10-fold higher than that towards arogenate, indicating that PPA-ATs predominantly catalyze the forward reaction, driving carbon flux from prephenate to arogenate [127,128]. However, suppression of PPA-AT through RNAi in *Petunia* petals resulted in a significant reduction in total PPA-AT activity, while not affecting aspartate aminotransferase activity. This suggests that the PPA-AT gene product is primarily responsible for converting prephenate to arogenate, with only a minor contribution to total aspartate aminotransferase activity in plants [127,128]. In *C. reinhardtii*, BLAST with a characterized sequence revealed one candidate, Cre02.g147302_4532.1, with predicted chloroplast localization (Table A1). Additionally, in *P. tricornutum*, one candidate for PPA-AT, XP_002176258.1, was predicted to localize in the chloroplast (Table A2).

#### 2.3.3. Prephenate and Arogenate Dehydratase

Prephenate dehydratase (PDT; EC 4.2.1.51) catalyzes the decarboxylation and dehydration of prephenate to phenylpyruvate, which is the second step in the cytosolic pathway. On the other hand, arogenate dehydratase (ADT; EC 4.2.1.91) converts arogenate to L-Phe, which is the final step in the plastid pathway. PDTs and ADTs are composed of two domains: a catalytic domain and a C-terminal ACT (aspartokinase, chorismate mutase, and TyrA) regulatory domain that is involved in the allosteric regulation by Phe. In plants, genes encoding monofunctional dehydratases are targeted to the plastids, as shown by localization studies [129]. Monocot and dicot plants consist of two or more isoforms of ADT, whereas green algae display a single gene [99]. In *C. reinhardtii*, a BLAST analysis revealed one candidate, Cre06.g261800_4532.1, with predicted chloroplast localization (Table A1). In *P. tricornutum*, one candidate for ADT, XP_002181766.1, was predicted to localize to both the cytosol and chloroplast. Similarly, one dual-function ADT/PDT candidate, EEC46980.1 (Phatr3_J3267), was also predicted to localize in both compartments (Table A2).

Recombinant ADTs from *Petunia* expressed in *E. coli* displayed strict or preferential substrate specificity toward arogenate over prephenate, leading to their classification as ADTs. Genetic studies revealed that RNAi suppression of ADT with strict arogenate specificity reduced L-Phe levels by approximately 80% in *Petunia* flowers [130]. This indicates that the plastid route is the predominant pathway for L-Phe biosynthesis in this plant organ. Additionally, a comparison of PPA-AT and ADT activities in *Petunia* petals revealed that PPA-AT activity is significantly higher than ADT activity by at least three orders of magnitude, suggesting that ADT may be the rate-limiting step in the plastidial L-Phe biosynthetic pathway [128,130].

All characterized ADTs possess an N-terminal transit peptide that targets the proteins to plastids. However, a dual subcellular localization of proteins can occur through the use of two alternative in-frame translation initiation codons. In such cases, the isoform translated from the first methionine possesses a plastid-targeting peptide, whereas a truncated isoform generated from the second methionine is likely targeted to the cytosol [130].

#### 2.3.4. Arogenate and Prephenate Dehydrogenase

Arogenate dehydrogenase (ADH, EC 1.3.1.43) and prephenate dehydrogenase (PDH, EC 1.3.1.12) catalyze the oxidative decarboxylation of arogenate and prephenate to produce L-Tyr and 4-hydroxyphenylpyruvate, respectively, using NAD+ or NADP+ as a cofactor (Figure 1). ADH and PDH enzymes are the major regulatory enzymes in Tyr biosynthesis, as they are competitively inhibited by L-Tyr and compete for the substrates also used in L-Phe biosynthesis. Moreover, PDHs lack an N-terminal chloroplast transit peptide and are cytosolic, like cytosolic CM. Interestingly, unlike plastid-localized ADHs, cytosolic PDHs lack sensitivity to allosteric inhibition by L-Tyr, despite their homology to plastidial ADH. Biochemical studies of *Arabidopsis* enzymes showed that ADH1 has strict substrate specificity toward arogenate, while ADH2 can also accept prephenate, but at three orders of magnitude lower catalytic efficiency than arogenate [61,62]. In *C. reinhardtii*, BLAST with a characterized sequence revealed two candidates, Cre06.g278350_4532.1 with predicted chloroplast localization and Cre06.g278350_4532.2 with cytosolic and chloroplast localization (Table A1). Additionally, in *P. tricornutum*, one candidate for PDH, XP_002177542.1, was predicted to localize to the plastid (Table A2).

ADH activity has been detected in various plant species, while PDH has been found in legumes [116,120]. Phylogenetic analyses of plant ADHs/PDHs showed that the legume PDH genes evolved through duplication of an ancestral plant ADH that probably occurred before the divergence of basal angiosperms, followed by neofunctionalization. Using a combined phylogenetic and structural approach of soybean ADH/PDH, researchers investigated how closely related enzymes, ADH and PDH, can be distinguished at the sequence level [69]. They identified a critical amino acid that controls the substrate specificity and L-Tyr sensitivity of ADH/PDH that underlies the functional evolution of alternative L-Tyr pathways in plants.

Specifically, they found that asparagine (Asn) at position 222 was specific to ADHs, i.e., absent in PDHs. Substituting the PDH amino acid at this position with an Asn was sufficient to convert PDH into ADH. Conversely, changing the critical Asn of an ADH into Cys altered the substrate specificity of the enzyme and converted it into a PDH. The researchers also demonstrated that the sequence requirement for PDH substrate recognition is less stringent. Importantly, this single amino acid change was linked to a simultaneous change in feedback regulation by L-Tyr, which inhibited ADHs but not PDHs [69].

#### 2.3.5. Phenylalanine Hydroxylase

L-Phe can be converted to L-Tyr by L-Phe hydroxylase (PheH, Figure 1). In protists and certain bacteria, Phe hydroxylation is catalyzed by iron-dependent monooxygenases that hydroxylate the aromatic ring of L-Phe to form L-Tyr. In plants, Phe hydroxylases are targeted to plastids and utilize 10-formyltetrahydrofolate as a cofactor, creating a unique link between folate and AAA metabolism in plants [131]. In *C. reinhardtii*, there is one candidate, Cre01.g029250_4532.1, with predicted chloroplast and extracellular localization (Table A1). Meanwhile, in *P. tricornutum*, one candidate for PheH, XP_002181086.1, was predicted to be secreted (Table A2).

#### 2.3.6. Phenylpyruvate and Tyrosine Aminotransferases

Phenylpyruvate aminotransferase (PPY-AT; EC 2.6.1.57) catalyzes the reversible transamination of phenylpyruvate to yield L-Phe, using PLP as a cofactor in the cytosol (Figure 1). The presence of PPY-AT has been recently validated by the elucidation of cytosolic L-Phe production in *Petunia*. Evidence of microalgae genes corresponding to similar activity has not been identified yet. However, in the *C. reinhardtii* database, nucleotide blast reveals no candidates for the *PPY-AT* gene, which could indicate the arogenate pathway for the biosynthesis of L-Tyr and L-Phe.

Similarly, L-Tyr aminotransferases (TAT; EC:2.6.1.5) interconvert 4-hydroxyphenylpyruvate (HPP) and L-Tyr. The forward reactions, PPY and Tyr transamination represent the final steps of the L-Phe and L-Tyr biosynthesis pathways, respectively.

Plants produce various L-Tyr-derived metabolites that are essential for plant adaptation and possess pharmaceutical and nutritional importance for human health. TATs catalyze the reversible reaction between L-Tyr and HPP, serving as the entry for both the biosynthesis of various natural products and the degradation of L-Tyr for energy and nutrient recycling. In most microbes, HPP is the intermediate of the L-Tyr biosynthetic pathway, and TATs are usually responsible for the final step of L-Tyr biosynthesis from HPP. Only a limited number of plant species (i.e., legumes) possess a microbial-like L-Tyr biosynthetic pathway via the HPP intermediate and likely have TAT enzymes that synthesize L-Tyr from HPP [69]. Most plants, however, synthesize L-Tyr through the alternative plastidial pathway [132]. As a result, in plants, TAT enzymes are likely responsible for the degradation and metabolism, rather than the biosynthesis, of L-Tyr. The current knowledge on the L-Tyr degradation pathway is mainly based on knowledge from microbes and mammals.

For cytosolic L-Tyr biosynthesis, an aminotransferase is necessary to complete this pathway, but no specific hydroxyphenylpyruvate aminotransferase (HPP-AT) has been identified. However, reversible TAT enzymes present in the cytosol of plant cells may have the potential to catalyze the Tyr-synthesizing reaction under certain conditions [133].

### 2.4. The Tryptophan Pathway

The plastidial biosynthetic pathway for L-Trp from chorismate was the first of the three AAAs to be fully elucidated in plants. Chorismate serves as a common precursor for at least four metabolic pathways, leading to the formation of L-Trp, L-Phe/L-Tyr, salicylate/phylloquinone, and folate. Hence, four enzymes from different pathways compete for chorismate, including CM, anthranilate synthase (AS; EC 4.1.3.27), isochorismate synthase (ICS; EC:5.4.4.2), and aminodeoxychorismate synthase (ADCS; EC:2.6.1.85) (Figure 1).

The L-Trp pathway consists of six enzymatic reactions that convert chorismate to L-Trp, with all of the involved enzymes localized in the plastids (Figure 1). Diatoms appear to have a similar L-Trp biosynthetic pathway to that of plants, beginning with the conversion of chorismate to anthranilate by eliminating the enolpyruvyl side chain through the transfer of the amino group from glutamine. AS is an amino-accepting chorismate–pyruvate lyase that catalyzes the first step in L-Trp biosynthesis, the formation of anthranilate. AS consists of large α and small β subunits (Asα and ASβ, respectively), which form an α/β heterodimer or an α2/β2 tetramer [72], Asα binds to chorismate and catalyzes the amination and pyruvate elimination reactions, whereas ASβ hydrolyses glutamine and provides ammonia to Asα [70]. A conformational change is evident when Asα encounters chorismate, leading to activation and ammonia transfer from Asβ. In times of excess L-Trp production, it blocks the conformational change of ASα and inhibits the activity of AS overall [68,70,134]. In *C. reinhardtii*, one candidate for Asα, i.e., Cre06.g306601_4532.1, has the predicted mitochondria localization, as per the software ‘WoLF PSORT II protein localization prediction tool’, and chloroplast localization, as per the software ‘ProtComp 9.0 protein localization prediction tool’, as well as one candidate for Asβ, i.e., Cre14.g620300_4532.1, with the predicted chloroplast localization via both software usages (Table A1). Additionally, for *P. tricornutum*, two candidates for AS, XP_002176337.1 and Phatr3_Jdraft1682, were predicted to localize to the plastid (Table A2).

Then, phosphoribosylanthranilate transferase (PAT, EC 2.4.1.18) shifts the phosphoribosyl part from phosphoribosylpyrophosphate to anthranilate and produces 5-phosphoribosylanthranilate. Phosphoribosylanthranilate isomerase (PAI, EC 5.3.1.24) catalyzes the third reaction with the irreversible rearrangement of 5-phosphoribosylanthranilate to 1-(o-carboxyphenylamino)-1-deoxy-ribulose 5-phosphate (CdRP), a reaction that can also occur non-enzymatically. In the next step, indole-3-glycerol phosphate synthase (IGPS, EC 4.1.1.48) catalyzes the irreversible conversion of CdRP to indole-3-glycerol phosphate. In *C. reinhardtii*, BLAST with a characterized sequence revealed one candidate for PAT, i.e., Cre10.g429150_4532.1; two candidates for PAI, i.e., Cre12.g519000_4532.1 and Cre12.g519000_4532.2; and one candidate for IGPS, i.e., Cre12.g528700_4532.1, with the predicted chloroplast localization for all candidates except IGPS, where it predicts mitochondrial localization, as per the software ‘WoLF PSORT II protein localization prediction tool’, and predicted chloroplast localization for all candidates via the software ‘ProtComp 9.0 protein localization prediction tool’ (Table A1). Additionally, for *P. tricornutum*, one candidate for PAT, XP_002182064.1, and one candidate for PAI, XP_002179396.1, were both predicted to have chloroplast localization (Table A2).

The final two reactions of the L-Trp pathway are catalyzed by the Trp synthase α subunit (TSα) and β subunit (TSβ, EC 4.2.1.20), respectively. TSα catalyzes the reversible retro-aldol cleavage of indole-3-glycerol phosphate to indole and glyceraldehyde 3-phosphate (G3P), and TSβ subsequently condenses indole and serine to produce L-Trp using pyridoxal 5-phosphate (PLP) as a cofactor. TSα and TSβ form an α2 β2 heterocomplex, and indole is transferred from the active site of TSα to that of TSβ through a 25-Å-long intermolecular tunnel. Whereas fungi possess a single gene encoding a bifunctional TSα-TSβ enzyme, bacteria such as *E. coli* have two separate genes encoding TSα and TSβ [68,135]. In *C. reinhardtii*, there are two candidates for TSα, i.e., Cre12.g528700_4532.1 and Cre12.g528700_4532.2, with the predicted cytosolic localization, and one candidate for TSβ, i.e., Cre03.g161400_4532.1, with the predicted mitochondria and chloroplast (Table A1). Additionally, in *P. tricornutum*, one candidate for TSα, XP_002176877.1, was predicted to be localized in the chloroplast, while one candidate for TSβ, XP_002182133.1, was predicted to be localized in the nucleus (Table A2). Elucidation of L-Trp biosynthesis in bacteria and plants is well-established, but our understanding of this pathway in microalgae remains significantly underexplored.

## 3. Metabolic Engineering for Enhancing AAAs in Microalga

Aromatic compounds play a vital role in the production of solvents, fine chemicals, food and feed additives, nutraceuticals, and pharmaceuticals [136,137]. The most common among these are phenol derivatives, phenylpropanoids, flavonoids, and alkaloids. However, the current supply of aromatic compounds largely depends on non-renewable energy sources, presenting a significant challenge due to their contribution to global climate change and the risk of resource depletion. To tackle this challenge, extensive research efforts have focused on developing engineered microbial strains with the capacity to produce a diverse array of chemicals and materials from renewable resources. In recent years, there has been significant progress in understanding the enzymes involved in the shikimate pathway, clarifying their functions and roles. Consequently, these enzymes have emerged as promising targets for inhibitors, leading to substantial research efforts aimed at their exploration and potential applications.

Efforts have been made over 20 years to optimize microbial processes for producing aromatic compounds from renewable feedstocks, involving pathway construction, enzyme engineering, metabolic flux modulation, and omics- and modeling-based technologies. Microorganisms like *Escherichia coli*, *Yarrowia lipolytica*, *Saccharomyces cerevisiae*, and *Corynebacterium glutamicum* are commonly used to produce aromatic compounds by integrating pathways or genetically modifying the hosts, benefiting from their rapid growth, scalability, and adaptability to various feedstocks. Based on the production of aromatic compounds from bacteria, common challenges associated with the production of aromatic compounds revolve around low precursor supply, feedback inhibition, and product cytotoxicity [138].

To tackle these problems, the following strategies for enhancing AAAs production have been applied; (i) increasing the availability of the direct precursors PEP and E4P [139,140]; (ii) replacing the native PTS-mediated glucose uptake system with alternative mechanisms [141,142,143]; (iii) enhancing the initial enzymatic reaction in the shikimate pathway to increase the yield of the enzyme DAHPS [144,145]; (iv) removing transcriptional and allosteric regulation [146]; (v) identifying and addressing rate-limiting enzymatic reactions [147]; (vi) preventing the diversion of carbon flux into competing pathways [148,149]; (vii) enhancing product export mechanisms; and (viii) preventing the degradation or reinternalization of the produced compounds [150]. Some examples of the metabolic-engineering strategies applied in microbial systems to enhance aromatic compound production have been listed in Table 5.

### 3.1. Possible Approaches That Can Be Applied to Enhance AAAs in Microalgae

#### 3.1.1. Increasing Precursors

In order to enhance the production of oil content, a CRISPRi-based technique was applied to inhibit the activity of one key enzyme, phosphoenolpyruvate carboxylase, which was responsible for partitioning carbon flux towards the tricarboxylic acid cycle in *C. reinhardtii*. This led to the availability of carbon for enhancing the acetyl-CoA supply, the main precursor for fatty acid production, and resulted in the enhancement of oil content by 18% [151]. Such strategies of enhancing precursors towards the desired pathway by applying molecular tools are common to various hosts [152]. Thus, a fundamental principle in the optimization of the shikimate pathway would be to enhance the availability of essential precursors, PEP and E4P. In addition, achieving a balanced supply of both precursors is of paramount importance for directing metabolic flux effectively into the shikimate pathway. In the microalgae *C. reinhardtii*, PEP is utilized by different pathways, such as glycolysis, gluconeogenesis, amino acid biosynthesis, the Calvin Benson Bassham (CBB) cycle, C4 and CAM (Crassulacean acid metabolism) photosynthesis, and fatty acid synthesis [153,154]. To increase the PEP pool, several useful strategies can be employed, including the overexpression of PEP-forming enzymes (i.e., pyruvate kinases and PEP carboxylase) and the inactivation of PEP-degrading enzymes (i.e., PEP carboxylase and PEP carboxykinase). However, the deactivation of PEP-degrading enzymes, while redirecting carbon flux towards the shikimate pathway, should be carefully carried out, as this led to a reduction in cell growth in *E. coli* [155].

#### 3.1.2. Modulation of Primary Metabolism

The primary metabolism in the eukaryotic system is complex and involves multiple transcription factors that control gene expression as a response to external stress [156]. The carbon flux shared by different biosynthetic pathways in the primary metabolism can be manipulated to target a specific desired pathway through transcription factors. Studies have been conducted on one such global regulatory protein, known as catabolite repressor/activator protein fruR in *E. coli,* where the inactivation of *fruR* enhanced the production of L-Tyr [157]. Also, when *fruR* was inserted into an L-Phe-producing strain of *E. coli* (PHE01) via the CRISPR/Cas-9 technique, one such resulting mutant, PHE07 (FruR^E173K^), had increased levels of specific production rates of L-Phe by approximately 37.79% and the yield by approximately 23.95% compared to the wildtype. Further transcriptomics and metabolomics of mutant PHE07 (FruR^E173K^) revealed upregulation of the genes involved in the gluconeogenesis pathway (*gpmM, pfkA, gapA,* and *pgk*), the pentose phosphate pathway (*zwf* and *tkt*), the Krebs cycle, and the glyoxylate shunt (*aceA*, *aceB*, *sucA*, and *sucD*), whereas the downregulation of genes was involved in the pyruvate metabolism pathway (*aceEF*, *acnB*, and *icd*), which resulted in the accumulation of PEP, which is a relevant precursor leading to biosynthesis of AAAs [149]. In pomegranates, overexpression of transcription factor PgMyb308-like in hairy roots enhanced the production of shikimate and AAAs [158]. The revelation of such genetic elements controlling the regulation of the primary metabolism in microalgae systems could be useful for enhancing the flux toward AAA production.

#### 3.1.3. Relieving Allosteric Control

Two major allosteric control points exist in the biosynthesis of AAAs, with the first at DAHPS in the shikimate pathway and the second at CM in the AAA biosynthesis pathway. In *E. coli*, there are three DAHP synthase isoenzymes, which are known as AroF, AroG, and AroH. Among these, AroG is the predominant contributor, accounting for approximately 80% of the total DAHP activity, while AroF and AroH make more modest contributions, representing around 15% and 5% of the overall DAHP synthase activity, respectively. Each of these three DAHP synthase isozymes is subject to feedback inhibition by a specific aromatic amino acid: L-Phe inhibits AroF, L-Tyr inhibits AroG, and L-Trp inhibits AroH. Given that AroG and AroF collectively contribute to over 95% of the total DAHP activity, engineering efforts have predominantly concentrated on these two enzymes. The point mutation of residues responsible for allosteric regulation has been a popular approach to release allosteric control and maintain enzyme activity. For instance, a single point mutation replacing leucine with glutamine at position 175 of AroG made the mutant insensitive to the allosteric regulation of DAHPS by L-Phe [159]. Another point mutation of Asn, replaced by Lys at position 8 of AroG, led to a Tyr-insensitive bacterial mutant for DAHPS [160]. Consequently, various feedback-resistant (fbr) enzyme variants have been engineered, including AroG mutants (Leu76Val, Asp146Asn, Pro150Leu, and Ser180Phe) [137,161] and AroF mutants (Pro148Leu and Gln152Ile) [162]. These engineered enzymes enable increased AAA yield by circumventing feedback inhibition. Another regulatory point exists at the chorismate branch, at which the chorismate mutase and prephenate dehydratase, encoded by *pheA* and *tyrA*, are feedback-regulated by the end products L-Phe and L-Tyr in *E. coli*. The most common strategy to overcome this bottleneck is the application of mutations that confer feedback resistance to chorismate mutase. Also, in yeast (*S. cerevisiae)*, the feedback inhibition was relieved by overexpression of the feedback-insensitive DAHP synthase (Aro3^fbr^ /Aro4^fbr^) and CM (Aro7^fbr^) [163]. A sequence comparison of bacterial *DAHPS* and *CM* with the microalgae enzymes could provide an idea about the allosteric residues, and site-directed mutagenesis could help create allosteric-insensitive mutants. A combination of engineered strains insensitive to both *DAPHS* and *CM* compared to only one gene-engineered strain could help elevate the levels of AAAs in microalgae as well.

#### 3.1.4. Overexpression of Feedback Insensitive Gene

Another idea could be the overexpression of allosterically insensitive *DAHPS* and *CM* genes from bacteria, yeast, or higher plants such as *A. thaliana* and *Petunia*. Plants have also been explored for strategies to enhance AAA levels as well. In the higher plant *A. thaliana,* a bacterial bifunctional *CM-PDT* gene that uses prephenate to generate phenylpyruvate was overexpressed in the plastid by removing the C-terminal allosteric domain, which canceled the feedback inhibition [164]. This resulted in transformants having enhanced levels of L-Phe- and L-Tyr-derived metabolites compared to L-Trp-derived metabolites. It indicates the utilization of branch point metabolite chorismate more by prephenate than anthranilate. Similarly, the feedback-insensitive *AroG_175_* gene that encoded for DAHPS from bacteria was transformed in a *Nicotiana tabacum* plant and revealed an enhanced level of L-Phe (43 fold), L-Tyr (24 fold), and L-Trp (10 fold) in leaves of transgenic plants as compared to control and wildtype [165]. Apart from *N. tabacum,* similar research on the overexpressing *AroG_175_* gene in *Solanum lycopersicum* [166] has resulted in enhanced downstream phenylpropanoids (volatile and non-volatile) and carotenoids. Also, overexpression of the *AroG_175_* gene in *Petunia* has resulted in an increase in the L-Phe-derived downstream metabolites involved in the floral volatiles benzenoid–phenylpropanoids (BPs) pathway [167]. A similar strategy could be applicable to microalgae as well. Although this strategy seems interesting, it is crucial to consider the overall metabolic consequences and metabolic cross-talks, such as in *Petunia hybrida.* Here, a cytosolic *CM* was overexpressed, and it resulted in a lower level of L-Phe in flowers and altered plastid development due to disrupted auxin metabolism because of excess phenylpyruvate production [168]. It would be interesting to learn how the overexpression of control point enzymes would affect the overall metabolite production in microalgae.

**Table 5:** Metabolic-engineering applications for enhancing AAAs and their derivatives.

Target	Organism	Strategy	Yield	Ref
Enhancing PEP level by replacing native PTS systems	*E. coli*	By combining a non-PTS sugar transport system with the overexpression of several crucial genes responsible for encoding DAHP synthase, transketolase, and chorismate mutase-prephenate dehydratase	Increased DAHP yield by 1.65 times higher than strain having PTS system L-Phe yield of 0.33 g/g glucose	[169]
Increasing the E4P pool to increase shikimic acid titer	*E. coli*	Increasing the E4P level by overexpression of the transketolase gene (*tktA*)	Increased shikimic acid titer from 38 to 52 g L^−1^	[170]
Prevent carbon loss and further boost E4P supply	*E. coli*	Enhance PEP and E4P supply by deletion of the gene *zwf1* (encoding glucose 6-phosphate dehydrogenase) and overexpression of gene tkl1 (encoding transketolase)	14.3 g L^−1^ L-Trp within 68 h in a fed-batch process from glycerol on a 15 L scale	[171]
Enhance the E4P and balance supply between E4P and PEP	*S. cerevisiae*	Increase the level of E4P by overexpression of transketolase (*Tkl1*) and ribose-5-phosphate ketol-isomerase (*Rki1*)	Increased the titer of shikimic acid by 25%	[172]
Increase shikimate production	*E. coli*	Integrate multiple strategies: feedback-resistant DAHP synthases; non-PTS glucose assimilation pathway was introduced to reinforce the supply of PEP; deletion of quinate dehydrogenase (QDH) to reduce the accumulation of quinate; inactivation of putative shikimate transporters ShiA and YdiN.	126.4 g/L of shikimate with a yield of 0.50 g/g glucose and a productivity of 2.63 g/L/h in a 30-L fermenter, highest reported titer	[173]
Increase AAA level	*E. coli*	Overcoming feedback inhibition by construction of a tunable switch by addition/starvation of different inducers and by replacement of feedback sensitive gene with a feedback-resistant (aroG^fbr^, trpE^fbr^, and pheA^fbr^)	0.32 g/L l-Trp, 0.60 g/L L-Phe, and 0.58 g/L l-L-Tyr	[161]
Increase AAA level	*S. cerevisiae*	Overcoming feedback inhibition by introducing feedback-insensitive DAHP synthase (Aro3^fbr^/Aro4^fbr^) and chorismate mutase (Aro7^fbr^)	4.5-fold increase of the flux through the AAA biosynthetic pathway	[174]
Increase AAA level	*Synechocystis*	Overcoming feedback inhibition by engineering strain expressing the aroG^fbr^ and tyrA^fbr^ genes from *E. coli*	903.8 ± 52.7 mg/gDW of L-Phe and 64.04 ± 3.67 mg/gDW of L-Tyr	[175]

## 4. Conclusions

With an increasing demand for sustainably produced pharmaceuticals, nutraceuticals, feed, food additives, etc., the focus is shifting towards a microalgae platform for industrial production of the aforementioned compounds, which is an immense need. Experimental research regarding the AAA biosynthesis pathway and its regulation in *C. reinhardtii* and *P. tricornutum* could be the first initiative toward understanding the native pathway limitations and employing metabolic-engineering strategies to overcome them. With fully sequenced genomes of both microalgae, characterization of the important enzymes in the AAA pathway, such as DAHPS, CM, ADH, and ADT could reveal significant regulatory mechanisms. With advanced genetic tools and techniques, enhancement of AAA production could be achieved, leading to the further development of these strains as a chassis for the production of high-value compounds.

## Figures and Tables

**Table 1:** Different applications for natural product accumulation and recombinant protein production in *C. reinhardtii*.

Product	Application	Yield	Strategy/Method	Ref
Lipids	Biofuel production	Oleic acid (C18:1) increased by 27.2%; total lipid accumulated up to 28% of dried biomass	CRISPR-Cas9 technology generated esterase/lipase/thioesterase *ELT1* knockout (Cre01.g00030) mutants (nuclear transformation via electroporation in strain CC-4349).	[29]
		4-fold increase in lipid content as compared to control	Severe iron deficiency induced triacylglycerols accumulation (modified media in strain CC-125).	[30]
		2.34-fold increase in lipid content; more than 80% of total SFA (saturated fatty acid) and MUFA (mono-unsaturated fatty acid) content	Nutrient starved (nitrogen, phosphorous), glucose supplementation (0.1%) increased lipid content and appropriate profile for biodiesel production (media modification in CC1010 strain).	[31]
Therapeutic protein: fourteenth human fibronectin type III domain, human vascular endothelial growth factor isoform121, and high mobility group protein B1	Pharmaceutical	2–3% of total soluble protein	Potential human therapeutic protein production (tenth human fibronectin type III domain, fourteenth human fibronectin type III domain, human vascular endothelial growth factor isoform121, and high mobility group protein B1) by recombinant DNA technology (chloroplast transformation by particle bombardment in strain 137c).	[32]
Antibodies: monoclonal antibody directed against a glycoprotein of the herpes simplex virus D (HSV8).	Biotechnological	High levels of protein accumulation	Expression of a large single chain coding sequence, IgA heavy chain protein fused to the light chain by a flexible linker peptide (chloroplast transformation by particle bombardment in strain 137c).	[33]
Antigens: HIV antigen P24	Biotechnological	0.25% of total soluble proteins (TSP)	Expression of codon-optimized the HIV antigen P24 gene variant (nuclear transformation via glass bead method in strain Elow47 and UVM11).	[20]
Carotenoids: CrtYB (phytoene–β-carotene synthase—PBS) gene Production of ‘Asthaxanthin’	Pharmaceutical	B-carotene:22.8 mg g^−1^ and Lutein: 8.9 mg g^−1^ up to 4.3 mg/L/day	Heterologous expression of phytoene–β-carotene synthase gene from red *yeast Xanthophyllomyces dendrorhous.* (nuclear transformation using chloroplast transit peptide via particle bombardment in strain CC-124). Synthetic redesign of ß-carotene ketolase gene, avoiding bottlenecking phytoene synthase and increasing activity of ß-carotene hydroxylase (Electroporation transformation in strain CC-125)	[8] [34]
Terpenoids (E)-α-bisabolene, the sesquiterpene biodiesel precursor	Sustainable energy production	10.3 ± 0.7 mg g^−1^ DCW of (E)-α-bisabolene 11.0 mg L^−1^) titer of (E)-α-bisabolene ((under light/dark cycle)	Overexpression of *Abies grandis* bisabolene synthase gene; downregulation of competing pathways via amiRNA knockdown and modified culture conditions (Glass bead transformation in strain UVM4)	[35]

**Table 2:** Exploration of *P. tricornutum* for natural product and recombinant protein production.

Product	Application of Products	Yield	Methods	Ref
Lipid	Biofuel	2.5-fold more lipid production, 57.8% DW	Overexpression of the endogenous *P. tricornutum* malic enzyme, transformed via electroporation.	[51]
		82% increase in the lipid production	Knockdown of pyruvate dehydrogenase kinase, transformed via electroporation.	[52]
		35% increase in neutral lipid accumulation, 76% increase in the valuable omega-3, eicosapentaenoic acid (EPA)	Overexpression of the endogenous diacylglycerol acyltransferase 2, transformed via electroporation.	[53]
		45-fold increase in triacylglycerol accumulation	Modification of the genome of the *P. tricornutum*, disruption of the UDP-glucose pyrophosphorylase gene using meganucleases and transcription activator-like effector nucleases.	[54]
		Enhanced total fatty acid (C18:0 and C18:1) content by 72%	Overexpression of *P. tricornutum* thioesterase, transformed using microparticle bombardment.	[55]
		Increased TAG content by 1.81-fold with a significant increase in polyunsaturated fatty acids	Overexpression of 1-acyl-sn-glycerol-3-phosphate acyltransferase designated AGPAT1, transformed via electroporation.	[56]
		43% increase in cellular lipid content	Knocked down the gene encoding for nitrate reductase, transformed via biolistic transformation.	[57]
		2–3-fold increase in TAG production	Overexpression of an endogenous type 2 diacylglycerol acyltransferase, transformed via biolistic transformation.	[58]
		Lipids increased by 30%, and 95% of the population changed the morphotype from fusiform to triradiate	Overexpression of a novel gene (*Pt2015*), transformed via biolistic transformation.	[59]
		Increased 23.19 and 25.32% in SFAs and between 49.02 and 54.04% in PUFAs	Overexpression of the endogenous *P. tricornutum* malic enzyme, transformed via biolistic transformation.	[60]
Geraniol	Pharmaceutical application (key intermediate in the biosynthesis of monoterpenoid indole alkaloids (MIAs))	Geraniol titer of 0.309 mg/L	Engineering *P. tricornutum* through extrachromosomal, episome-based expression for the heterologous biosynthesis of geraniol, transformed by bacterial conjugation.	[41]
Plant triterpenoids (Betulin, Lupeol)	Pharmaceutical application (antiprotozoal, antimicrobial, antitumor, precursor for the treatment of certain cancers and HIV)	Successful production of betulin and its precursor lupeol (0.1 mg/L over 2 days of culturing).	Introducing three plant enzymes in *P. tricornutum*: a *Lotus japonicus* oxidosqualene cyclase (lupeol synthase) and a *Medicago truncatula* cytochrome P450 along with its native reductase, transformed via biolistic transformation.	[12]
CBGA	Pharmaceutical application (precursor to several cannabinoids (CB) such as well-known cannabidiol (CBD) and delta-9-tetrahydrocannabinol (THC))	Production of cannabigerolic acid (CBGA) up to 4.1 (±0.2) mg/kg of microalgae fresh biomass weight.	Engineering *P. tricornutum* to express a mutant version of the *Streptomyces* sp. NphB, a non-cannabis aromatic prenyltransferase enzyme, either by random integrated chromosomal expression (RICE) or extrachromosomal expression (EE).	[40]
Olivetolic acid (OA)	Pharmaceutical application (cannabinoid precursor)	Successful integration and functionality of the heterologous cannabis genes TKS and OAC, and significant olivetolic acid accumulation (0.6–2.6 mg/L).	Engineering *P. tricornutum* through the introduction of *C. sativa* tetraketide synthase and olivetolic acid cyclase, olivetolic acid. Transformed via bacterial conjugation.	[61]
Antibodies	Pharmaceutical application (Hepatitis B vaccine)	Antibody concentration about 8.7% of total soluble protein, which complies with 21 mg antibody per gram algal DW or 400 mg antibody in a 250 mL culture.	Heterologous expression of a fully assembled human IgG antibody against Hepatitis B surface antigen in *P. tricornutum,* transformed with biolistic transformation.	[50]
Polyhydroxybutyrate (PHB)	Biodegradable plastics	Sufficient production in PHB levels of up to 10.6% of algal DW.	Introducing the bacterial PHB pathway of *R. eutropha* H16. The enzymes PhaA (ketothiolase), PhaB (acetoacetyl-CoA reductase), and PhaC (PHB synthase) were expressed with stable nuclear transformation.	[49]

**Table 3:** Kinetic parameters values for DAHPs across microbial and plant systems.

Species	Isozymes	Inhibitors	Substrate	*K*m [mM]	Ref
*E. coli*	AroG	Phe	PEP E4P	0.08 0.9	[82]
	AroF AroH	Tyr Trp	PEP E4P	0.013 0.0814	[83]
*S. cerevisiae*	Aro3	Phe	PEP E4P	0.018 0.13	[84]
	Aro4	Tyr	PEP E4P	0.125 0.5	[85]
*Mycobacterium tuberculosis*	mtDAHPS	Phe	PEP E4P	0.025 0.037	[86]
*Arabidopsis thaliana*	DHS1	Chorismate; Caffeate	PEP E4P	0.25 2.842	[78]
	DHS2	Tyr, Trp, Chorismate; Caffeate	PEP E4P	0.36 1.755	
	DHS3	Chorismate; Caffeate	PEP E4P	0.706 1.55	

PEP, phosphoenol pyruvate; E4P, erythrose-4-phosphate; Phe, phenylalanine; Tyr, tyrosine; Trp, tryptophan.

**Table 4:** Comparison of steady-state kinetic parameters of chorismate mutases across various organisms.

Enzyme	Species	*k_cat_* (s^−1^)	*K_m_* (mM)	*k_cat_*/*K_m_* (M/s)	Ref
***At*** **CM1**	*Arabidopsis thaliana*	16	0.55	29,090	[108]
***At*** **CM2**	*Arabidopsis thaliana*	39	0.15	260,000	[108]
***At*** **CM3**	*Arabidopsis thaliana*	13	1.10	11,818	[108]
***Ph*** **CM1**	*Petunia hybrida*	25	0.174	143,678	[122]
***Ph*** **CM2**	*Petunia hybrida*	64	0.009	7,136,000	[122]
***Pp*** **CM1**	*Physcomitrella patens*	21	2.39	8660	[111]
***Pp*** **CM2**	*Physcomitrella patens*	19.5	2.33	8370	[111]
***Sm*** **CM**	*Selaginella moellendorffii*	18.8	5.19	3620	[111]
***Sc*** **CM**	*Saccharomyces cerevisiae*	360	3.8	94,736	[123]
***Hp*** **CM**	*Hansenula polymorpha*	319.1	16.7	19,107	[124]
***An*** **CM**	*Aspergillus nidulans*	82	2.3	35,652	[125]
***PpCM1***	*Pinus pinaster*	29.4	1.6	18.4	[126]
***PpCM2***	*Pinus pinaster*	35	1.7	20.6	[126]

## Data Availability

No new data were created or analyzed in this study.

## Appendix A

The appendix section contains detailed information about the genes involved in the shikimate pathway and aromatic amino acid (AAA) biosynthesis in *Chlamydomonas reinhardtii* and *Phaeodactylum tricornutum*.

Table A1: transcript IDs of all the genes involved in the shikimate pathway and AAA biosynthesis in *C. reinhardtii*, along with their predicted subcellular localization.

Table A2: transcript IDs of all the genes involved in the shikimate pathway and AAA biosynthesis in *P. tricornutum*, along with their predicted subcellular localization.

These tables provide a foundational reference for understanding the metabolic organization and localization of these pathways in these model organisms.

**Table A1:** List of transcript IDs of all the genes involved in shikimate pathway and AAA biosynthesis, along with its predicted localization in *C. reinhardtii*.

ID	Transcript ID	Predicted Sub-Cellular Localization	Reference
DAHP synthase	Cre17.g726750_4532.1 Cre17.g726750_4532.2 Cre17.g726750_4532.3	Chloroplast ^a,b^	[18,176]
DHQS	Cre08.g368950_4532.1 Cre08.g368950_4532.2	Chloroplast ^a,b^ Cytosol ^a,b^	
DHD/SDH	Cre08.g380201_4532.1	Chloroplast ^a,b^	
SK	Cre10.g436350_4532.1 Cre10.g436350_4532.2	Chloroplast ^a^ Extracellular (Secreted) ^b^	
EPSPS	Cre03.g181300_4532.1	Chloroplast ^a,b^,	
CS	Cre03.g145747_4532.1 Cre03.g145747_4532.2	Mitochondria ^a^ Chloroplast ^a,b^	
CM	Cre03.g155200_4532.1 Cre03.g155200_4532.2 Cre03.g155200_4532.3	Chloroplast ^a,b^, Cytosol ^b^	
PPA-AT	Cre02.g147302_4532.1	Chloroplast ^a,b^	
ADT/PDT	Cre06.g261800_4532.1	Chloroplast ^a,b^	
ADH/PDH	Cre06.g278350_4532.1 Cre06.g278350_4532.2	Chloroplast ^a,b^ Cytosolic ^a^, Chloroplast ^b^	
Phe hydroxylase	Cre01.g029250_4532.1	Chloroplast ^a^ Extracellular (Secreted) ^b^	
Asα	Cre06.g306601_4532.1	Mitochondria ^a^, chloroplast ^b^	
Asβ	Cre14.g620300_4532.1	Chloroplast ^a,b^	
PAT	Cre10.g429150_4532.1	Chloroplast ^a,b^	
PAI	Cre12.g519000_4532.1 Cre12.g519000_4532.2	Chloroplast ^a,b^	
IGPS	Cre12.g528700_4532.1	Mitochondria ^a^ Chloroplast ^b^	
TSα	Cre12.g528700_4532.1 Cre12.g528700_4532.2	Cytosol ^a,b^	
TSβ	Cre03.g161400_4532.1	Mitochondria ^a^ Chloroplast ^b^	
Cationic amino acid transporter	Cre07.g329050_4532.1 Cre07.g329050_4532.2 Cre01.g041050_4532.1 Cre01.g041050_4532.2 Cre01.g041050_4532.3 Cre01.g041050_4532.4	Plasma membrane ^a,b^ Vacuole ^a^, plasma membrane ^b^ Mitochondria ^a^, plasma membrane ^b^ Chloroplast ^a^, Vacuole ^b^, plasma membrane ^b^ Chloroplast ^a^, Vacuole ^b^, plasma membrane ^b^ Cytoplasm ^a^, extracellular ^b^	
Amino acid transporter transmembrane	Cre16.g801997_4532.1	Nucleus ^a^, Secreted ^b^	

^a^ = WoLF PSORT II protein localization prediction tool; ^b^ = ProtComp 9.0 protein localization prediction tool.

**Table A2:** List of transcript IDs of all the genes involved in shikimate pathway and AAA biosynthesis, along with its predicted localization in *P. tricornutum*.

ID	Transcript ID	Location	Ref
DAHPS	XP_002177054.1	Chloroplast ^ab^	[44]
DHQS	XP_002180805.1 Phatr3_J20809	Chloroplast ^ab^	[44]
DHQ/SDH	XP_002179655.1	Chloroplast ^a^, Secreted ^b^	[44]
SK	XP_002184173.1 Phatr3_J6807	Chloroplast ^a^, Secreted ^b^	[44]
EPSPS	XP_002178032.1	Chloroplast ^ab^	[44]
CS	XP_002177933.1 Phatr3_J43429	Chloroplast ^a^, Secreted ^b^	[44]
CM	Phatr3_draftJ417	Chloroplast ^a^, cytosol ^b^	[57]
CM	Phatr3_J43277	Chloroplast ^a^, cytosol ^b^	[57]
PPA-AT	XP_002176258.1	Chloroplast ^ab^	[44]
PPY-AT	XP_002186145.1	Chloroplast ^a^, secreted ^b^	[44]
ADT	XP_002181766.1	Cytosol ^a^, chloroplast ^b^	[44]
ADT/PDT	EEC46980.1 Phatr3_J3267	Cytosol ^a^, chloroplast ^b^	[44]
PDH	XP_002177542.1	Plastid ^ab^	[44]
PheH	XP_002181086.1	Secreted ^b^, nucleus ^a^	[44]
AS	XP_002176337.1 Phatr3_Jdraft1682	Plastid ^a^, extracellular ^b^	[44]
PAT	XP_002182064.1	Chloroplast ^ab^	[44]
PAI	XP_002179396.1	Chloroplast ^a^, extracellular ^b^	[44]
IGPS	AAL79536.1	Chloroplast ^ab^	[177]
TS	XP_002176877.1	Chloroplast ^ab^	[44]
TSβ	XP_002182133.1	Nucleus ^ab^	[44]
Cationic amino acid transporter	B7G8I9_PHATC	Membrane ^b^, plastid ^a^	[44]
Amino acid transporter transmembrane	B7GA51_PHATC	Secreted ^b^, plastid ^a^	[44]

^a^ = WoLF PSORT II protein localization prediction tool; ^b^= ProtComp 9.0 protein localization prediction tool.
